# Sandfly Fever Sicilian Virus, Algeria

**DOI:** 10.3201/eid1405.071487

**Published:** 2008-05

**Authors:** Arezki Izri, Sarah Temmam, Grégory Moureau, Boussad Hamrioui, Xavier de Lamballerie, Rémi N. Charrel

**Affiliations:** * Université Paris 13, Bobigny, France; † Université de la Méditerranée, Marseille, France; ‡ Université d’Alger, Alger, Algeria

**Keywords:** phlebovirus, sandfly, arbovirus, human, Toscana, dispatch

## Abstract

To determine whether sandfly fever Sicilian virus (SFSV) is present in Algeria, we tested sandflies for phlebovirus RNA. A sequence closely related to that of SFSV was detected in a *Phlebotomus ariasi* sandfly. Of 60 human serum samples, 3 contained immunoglobulin G against SFSV. These data suggest SFSV is present in Algeria.

Recent attention has been drawn to *Toscana virus* (family *Bunyaviridae*, genus *Phlebovirus,* species *Sandfly fever Naples virus*) in countries surrounding the Mediterranean because the virus causes meningitis during summer. *Sandfly fever Sicilian virus* (SFSV) is a distinct arthropod-borne phlebovirus transmitted by sandflies, specifically by *Phlebotomus papatasi* (*1*). It was discovered in Italy (Palerma, Sicilia), where it affected troops of the World War II Allied Army Forces after the Sicily landings in 1943. SFSV infection produces a febrile illness during the warm season; in contrast with Toscana virus infection, SFSV infection is not associated with neurologic manifestations.

Human cases of SFSV infection have been reported from Italy, Egypt, Pakistan, Iran, and Cyprus (*1*,*2*). Seroprevalence studies performed with human or vertebrate serum indicate that SFSV, or a closely related virus, is circulating in Jordan (*3*), Israel (*4*), Sudan (*5*), Tunisia (*6*), Pakistan (*7*), Egypt (*8*), Bangladesh (*9*), and Iran (*10*). The most comprehensive study, initiated by Tesh et al. (*11*), did not find neutralizing antibodies reactive to SFSV in human serum from the Algerian populations of Tamanrasset and Djanet. Therefore, at the outset of this study, no evidence or data suggested the presence of SFSV in Algeria.

## The Study

**Figure 1:**
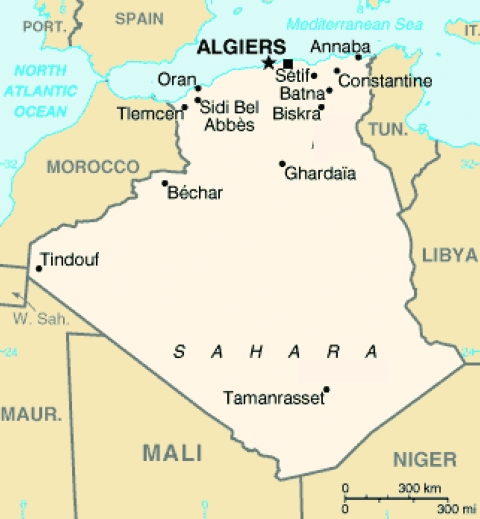
Map of Algeria showing where sandflies were trapped (■).

Over a 4-night period in July 2006, a total of 460 sandflies were trapped as described (*12*). Trapping was performed at Larbaa Nath Iraten (previously known as Fort National) in the Kabylian region of Algeria, near Tizi Ouzout (Figure 1). CDC Miniature Light Traps were adapted for sandfly capture by using an ultrafine mesh. Traps were hung 1–2 m above ground. They were placed during late afternoon in or near animal housing facilities (chickens, rabbits, goats, horses). Each morning, sandflies were collected, identified morphologically, and placed in 1.5-mL microfuge tubes. Captured sandflies belonged to 7 species: *P. perniciosus* (n = 364), *P. longicuspis* (n = 61), *P. sergenti* (n = 21), *P. ariasi* (n = 6), *P. perfiliewi* (n = 3), *P. papatasi* (n = 1), and *Sergentomyia minuta* (n = 1). They were organized into 24 pools, each containing up to 30 sandflies. Each pool was ground in RNA NOW chaotropic solution (Ozyme, Montigny le Bretonneux, France). RNA purification was performed according to the manufacturer’s protocol. A total of 10 μL of RNA suspension was used for reverse transcription with random hexanucleotide primers with the Taqman Reverse Transcription Reagents (Applied Biosystems, Foster City, CA, USA) in a final volume of 50 μL, according to the manufacturer’s recommended protocol. To test these specimens for Toscana virus RNA and phlebovirus RNA, we used 10 μL of cDNA in the previously described assays (*12*,*13*). Pool F tested positive with nested primers Phlebo2+/Phlebo2–. The PCR product was sequenced directly with primers used for PCR amplification. A 201-nt sequence (excluding primers) was obtained and submitted to the National Center for Biotechnology Information BLAST program, which retrieved a unique hit, consisting of Cyprus phlebovirus polymerase gene with a 92% identity score. Because *Cyprus virus* has been reported to be closely related to SFSV (*2*), we amplified and sequenced the homologous genome region of SFSV strain Sabin by using the primers described above. The same approach was applied to *Arbia virus,* a related phlebovirus isolated in Italy simultaneously with Toscana virus for comparative analysis. These 3 sequences were deposited in the GenBank database under accession nos. EU240880, EU266619, and EU266620. Laboratory contamination can be excluded because the sequence corresponding to SFSV-Algeria is divergent from its closest sequence (SFSV-Italy-Sabin) by 4%, which corresponds to 8 nt mutations; in addition, SFSV-Italy-Sabin has been manipulated after PCR amplification and sequencing of SFSV-Algeria to compare it genetically with the sequence obtained from Algerian sandflies.

**Figure 2:**
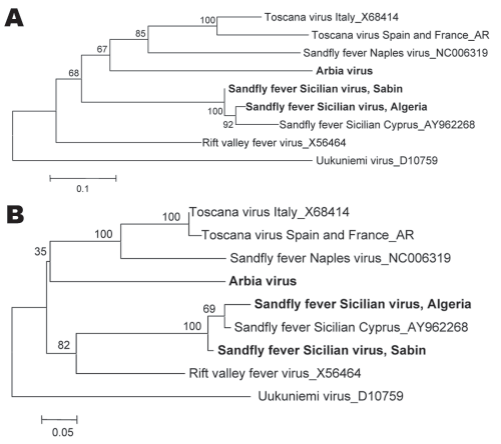
Phylogenetic analysis of *Sandfly fever Sicilian virus,* Algeria, based on A) 207-nt or B) 67-aa sequence in the polymerase gene. Distances and groupings were determined by the pairwise or Kimura 2-parameter algorithm and neighbor-joining method with the MEGA v2 software program (www.megasoftware.net). Bootstrap values are indicated and correspond to 500 replications. **Boldface** indicates virus names that correspond to sequences determined in this study. Scale bars indicate pairwise nucleotide distances (0.1 = 10%) and Kimura 2-parameter amino acid distances (0.05 = 5%).

Together with homologous sequences of selected phleboviruses, the 3 sequences determined in this study were used to perform genetic distance comparison and phylogenetic analysis. Nucleotide and amino acid distances are presented in the Table. Distance analysis unambiguously indicated that Algeria virus is a variant genotype of SFSV. The same conclusion applied to Cyprus phlebovirus. These 3 viruses exhibited amino acid and nucleotide distances of <9.3% and <7.5%, respectively. Phylogenetic analyses (Figure 2) indicated that Algeria formed a strong cluster (100% bootstrap support) with SFSV strain Sabin and the Cyprus phlebovirus. Therefore, we propose that they can be considered as 3 variant strains of the tentative species SFSV. Because sandfly material was stored in a chaotropic solution, virus isolation was not possible, which will necessitate field work with storage conditions suitable for virus isolation attempts.

**Table:** Pairwise genetic distances between Sandfly fever Sicilian virus sequence and homologous sequences of selected phleboviruses* View Actual Table

No.	Virus	1	2	3	4	5	6	7	8	9
		Nucleotide sequences								
1	Sandfly fever Sicilian virus, Algeria	–	7.5	4.0	40.4	38.8	42.3	40.1	38.5	48.2
2	Sandfly fever Sicilian virus, Cyprus	4.5	–	6.5	42.7	41.3	39.3	43.3	38.5	53.0
3	Sandfly fever Sicilian virus, Italy	9.3	1.5	–	39.1	37.9	42.3	39.6	35.8	46.5
4	Arbia virus	54.7	52.2	50.7	–	38.2	42.2	40.9	40.4	53.8
5	Toscana virus, Italy	48.0	47.8	44.0	42.7	–	17.4	30.4	38.1	48.2
6	Toscana virus, France	46.3	47.8	47.8	44.8	1.5	–	34.8	42.0	52.0
7	Sandfly fever Naples virus, Sabin	52.0	50.7	48.0	52.0	25.3	25.4	–	41.2	51.3
8	Rift Valley fever virus	41.3	37.3	34.7	45.3	37.3	41.8	46.7	–	47.1
9	Uukuniemi virus	69.3	67.2	61.3	64.0	60.0	65.7	62.7	58.7	–
		Amino acid sequences								

*Sandfly fever Sicilian virus isolated from *Phlebotomus ariasi* sandflies in Algeria. Selected phleboviruses are those for which the corresponding sequence was available in the GenBank database.

Detection of SFSV RNA in sandflies led us to test human serum for SFSV antibodies. We tested 60 samples from healthy persons for SFSV immunoglobulin (Ig) G by indirect immunofluorescence assay as described (*14*) with minor modifications. Briefly, equal quantities of infected and uninfected Vero cells were mixed together and spotted onto 2-well glass slides through a 3-min cytospin-based centrifugation at 900 rpm. Samples were tested at a 1:20 dilution in phosphate-buffered saline. Three (5%) samples contained SFSV IgG but not Toscana virus IgG.

## Conclusions

Together, molecular and serologic data constitute evidence that SFSV is present in Algeria. Genetic analysis of a partial region of the polymerase gene (L genome segment) indicated that the Algerian, Italian, and Cypriot strains of SFSV are closely related. Another study performed with M RNA sequences showed that Italian and Cypriot strains of SFSV are closely related as well (*15*).

To our knowledge, SFSV has been previously isolated in *P. papatasi* flies only. In this study, detection of SFSV RNA in 1 female *P. ariasi* sandfly must be interpreted with caution. In particular, this finding does not mean that *P. ariasi* is a vector of SFSV in the study region. The presence of SFSV RNA may result from mechanical transmission from a viremic vertebrate. Therefore, specific studies should be conducted to investigate vectors of SFSV in Algeria. Seroprevalence data demonstrate that SFSV or an SFSV-related virus can infect humans. Further studies are needed to determine whether the clinical picture is limited to self-resolving febrile illness, as previously reported in Italy.
